# Evolving paradigms in prostate cancer screening: a decade of bibliometric insights and technological advancements

**DOI:** 10.3389/fonc.2025.1511880

**Published:** 2025-02-25

**Authors:** Keqiang Chai, Changhong Xu, Jiangwei Man, Yun Deng, Li Yang

**Affiliations:** ^1^ Department of Urology, The Third Affiliated Hospital of Gansu University of Chinese Medicine, Baiyin, Gansu, China; ^2^ Department of Urology, Institute of Urology, Gansu Province Clinical Research Center for Urinary System Disease, Lanzhou University Second Hospital, Lanzhou, Gansu, China

**Keywords:** prostate cancer screening, bibliometric analysis, precision medicine, diagnostic technologies, risk stratification

## Abstract

**Objective:**

Prostate cancer is a major threat to global male health. This study uses bibliometric methods to analyze the dynamics and trends in prostate cancer screening research, with the aim of optimizing screening strategies and informing policy decisions.

**Methods:**

Utilizing the Web of Science Core Collection database, this study retrieved prostate cancer screening-related literature published between 2014 and 2024, totaling 5,409 articles. Data processing and analysis were conducted using CiteSpace and the Bibliometrix R package, including citation network analysis, co-word analysis, cluster analysis, and trend analysis.

**Results:**

The analysis revealed the following key findings: (1) Global literature on prostate cancer screening has grown annually, with the United States, Europe, and China leading research activity; (2) Research hotspots include the risks and benefits of prostate-specific antigen (PSA) testing, MRI-based screening technologies, and the use of molecular and genetic biomarkers; (3) Emerging technologies, such as machine learning and nanodiagnostic techniques, are enhancing diagnostic precision and reducing overdiagnosis; (4) Network analysis of collaborations reveals a shift toward transnational and interdisciplinary research, particularly in integrating biomedical and computer science to drive rapid advancements in screening technologies.

**Conclusion:**

This study confirms the ongoing vibrancy and technological advancement in the global field of prostate cancer screening research, emphasizing the trend towards precision medicine. Future development of prostate cancer screening strategies should focus on risk-adapted screening and the application of novel biomarkers to optimize screening outcomes and reduce unnecessary medical interventions.

## 1 Introduction

As the global population ages, prostate cancer has become a significant disease impacting men’s health worldwide. According to data from the World Health Organization, prostate cancer is the second most common type of cancer among men globally, with both its incidence and mortality rates on the rise (1, 2). Early detection and treatment of prostate cancer have significantly enhanced patient survival rates, highlighting the importance of developing and implementing effective screening strategies (3, 4).

Since the late 1980s, the Prostate-Specific Antigen (PSA) test has been a cornerstone for prostate cancer screening (5). This test aids in the diagnosis of prostate cancer through the measurement of PSA levels in the blood (6). While the PSA test has facilitated early detection of prostate cancer, its use remains controversial. Criticisms focus on overdiagnosis and overtreatment, particularly of indolent cancers, which can lead to unnecessary medical interventions (7, 8). Additionally, the specificity and sensitivity of PSA screening are limited, and the issues of misdiagnosis and missed diagnoses cannot be overlooked (9). These issues may result in unnecessary biopsies and treatments, or miss the optimal timing for treating early-stage cancers.

In this context, this study introduces bibliometric methods to provide a novel perspective and tool for analyzing the research dynamics and developmental trends of prostate cancer screening. Bibliometrics, a discipline that applies mathematical and statistical methods to analyze scientific literature, can systematically assess the volume of literature, growth trends, major research institutions and scholars, and research themes and hot topics within a field (10, 11). Through the bibliometric analysis of extensive literature data, this study aims to comprehensively map the knowledge structure of the prostate cancer screening field, identify research hotspots and key changes, and explore their impact on clinical practice and policy-making.

Specifically, this research utilizes bibliometric tools such as citation analysis, co-word analysis, cluster analysis, and trend analysis to conduct a comprehensive analysis of prostate cancer screening literature over the past decade. We anticipate that these analyses will not only reveal the main research trends and themes in the field but also provide a scientific basis for optimizing prostate cancer screening strategies and offer insights for formulating global male health policies. Moreover, this study will assess the contributions and impacts of different countries and research institutions, as well as their collaboration patterns in global prostate cancer screening research.

By deeply analyzing the current state and evolution of global prostate cancer screening research, this paper aims to provide valuable insights for global health policymakers and medical professionals, thereby fostering the development of more effective and precise cancer screening strategies.

## 2 Methods

### 2.1 Data source and selection

This study utilized the Web of Science Core Collection database, focusing on the fields of Title, Abstract, and Keywords for retrieving scientific literature on prostate cancer screening. The search string used was: “Prostate Cancer” OR “Prostatic Neoplasms” AND “Screening” OR “Early Detection” OR “Diagnosis”, with a cut-off date of June 30, 2024. Only peer-reviewed original research articles were selected, excluding reviews, conference papers, and non-English publications, totaling 5,409.

### 2.2 Data processing

The downloaded records were stored in plain text format and cleaned using CiteSpace6.2R6 bibliographic management software. The exclusion criteria for records included missing essential information such as authors, keywords, or abstracts, and duplicates or inconsistencies were systematically removed to ensure the accuracy of subsequent analyses.

### 2.3 Analytical tools

The study utilized CiteSpace for citation network analysis, co-word analysis, and cluster analysis with specific parameters set to refine our insights. Years per slice were set to 1 year to focus on annual trends, and the scale factor k was set to 5 to optimize the visibility and separation of nodes. For visualization purposes, labels were assigned based on the frequency of appearance, typically selecting the top 10-20 items for annotation to highlight the most significant trends and nodes within the research field. The Bibliometrix R package was employed for comprehensive descriptive statistical analysis, further assessing trends in literature production, distribution by country/region, and major journal and keyword frequencies, ensuring a robust analytical framework for our bibliometric study.

### 2.4 Analysis steps descriptive statistical analysis

Using the Bibliometrix R package, an annual publication trend analysis was performed on all selected documents to identify active periods in prostate cancer screening research. Contributions from different countries/regions to prostate cancer screening research were also analyzed, along with the most active institutions and scholars in this field.

### 2.5 Journal and article impact analysis

The journals that published the most articles on prostate cancer screening were identified, and their impact factors and Chinese Academy of Sciences (CAS) divisions were analyzed to assess the academic influence of the research findings. Additionally, high-impact articles within the research field were determined through citation analysis.

### 2.6 Keywords analysis

Keywords were analyzed using co-word analysis with CiteSpace software, which involved constructing a keyword co-occurrence network diagram to reveal research hotspots and the evolution of the knowledge structure. This analysis helped identify the main research themes and trends within the field.

### 2.7 Citation network analysis

Citation network analysis was conducted using CiteSpace to explore key and foundational literature in prostate cancer screening research, marking significant milestones in the development of this field.

### 2.8 Research trends and prospects

Based on the results of the aforementioned analyses, future research trends in prostate cancer screening over the coming years were predicted using time series forecasting models.

### 2.9 Data visualization

All analytical results were visualized through graphs and tables to facilitate a more intuitive presentation of the research findings. These visual representations included, but were not limited to, annual publication trend graphs, national/regional contribution charts, keyword co-occurrence network diagrams, and citation network graphs.

## 3 Results

### 3.1 Scientific output

**Figure 1:**
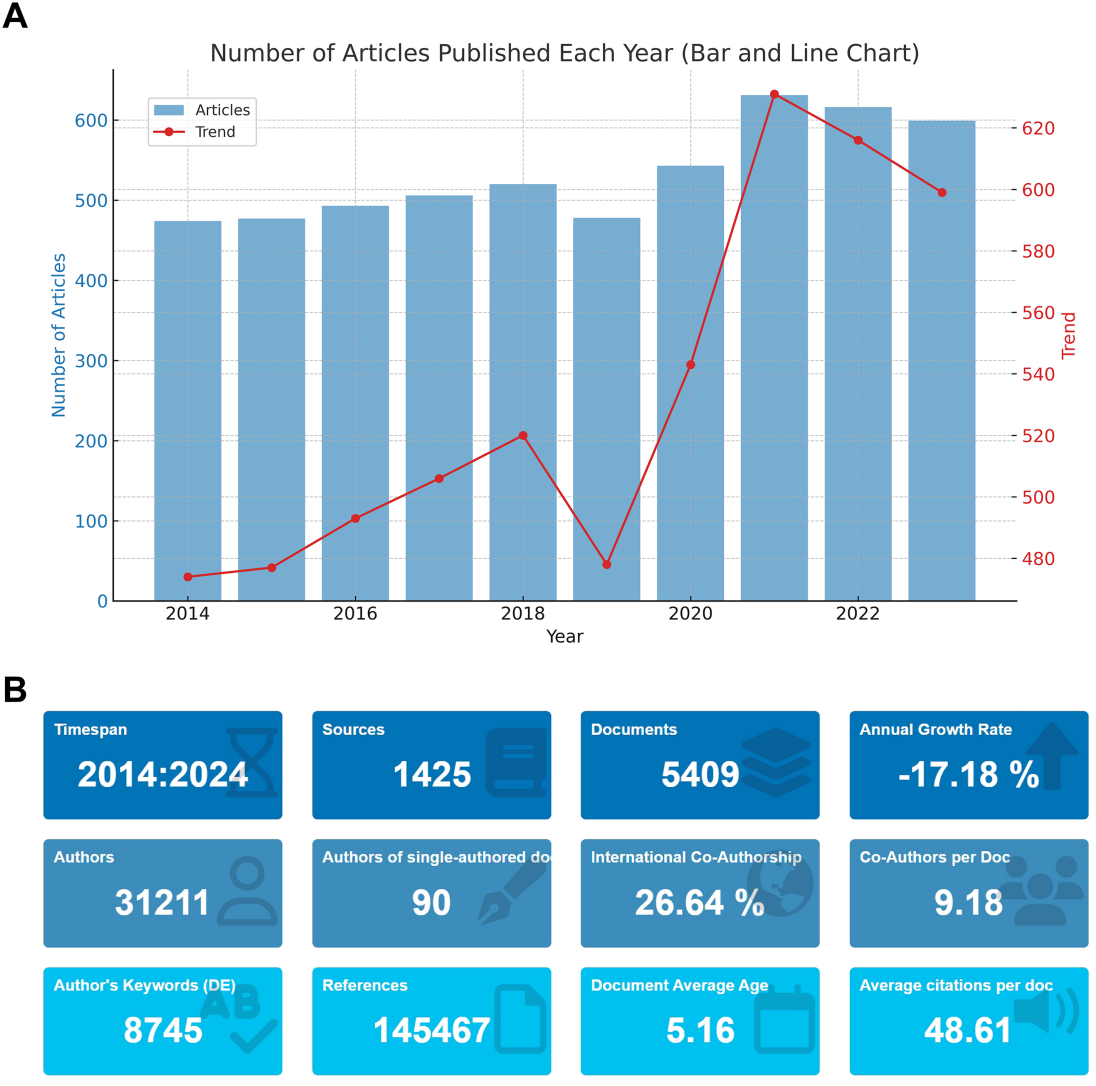
Scientific Output in Prostate Cancer Screening from 2014 to 2024. **(A)** Annual publication trends in prostate cancer screening research from 2014 to 2024. **(B)** Overview of the deduplicated literature data, including the number of articles, journals, authors, and citations.

**Figure 1A** indicates a steady increase in the number of papers published in the field of prostate cancer screening, with a peak of 631 publications in 2019, possibly influenced by the global COVID-19 pandemic. Additionally, **Figure 1B** presents the overall statistics of the literature data after deduplication. These 5,409 articles stem from 1,425 journals, involving 31,211 collaborating authors. The rate of international collaboration reached 26.64%, with a total of 145,467 references cited. This extensive engagement highlights the dynamic and globally interconnected nature of research in prostate cancer screening.

### 3.2 Authors

**Figure 2:**
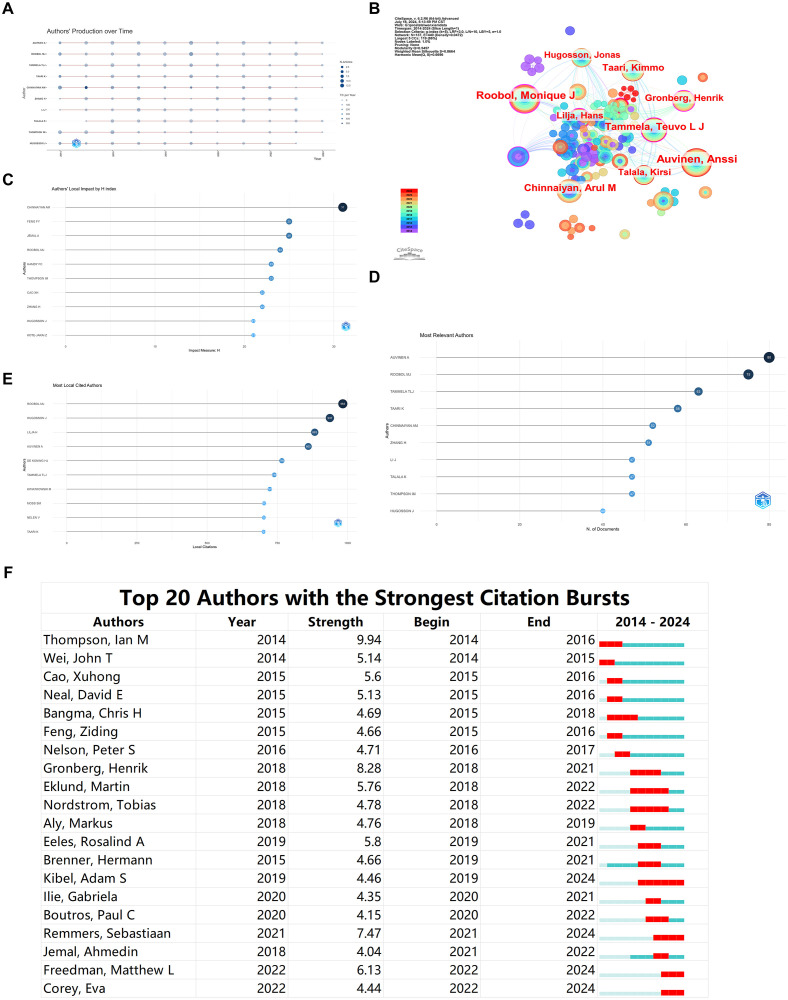
Bibliometric Visualization of Author Distribution in Prostate Cancer Screening. **(A)** Annual publication count for the top ten authors in prostate cancer screening from 2014 to 2024. **(B)** Author collaboration network visualization. **(C)** Local impact of authors based on H index. **(D)** Ranking of the most relevant authors. **(E)** Citation ranking of authors. **(F)** Burst analysis of emerging authors.

**Figure 2A** illustrates the annual publication count by the top ten authors in the field from 2014 to 2024, showing an increase in the number of papers published by these authors since 2019. The authors’ collaboration network comprises 137 nodes and 440 connections, with a network density of 0.0472. The node size represents the number of projects, while different colors indicate different years. The lines between nodes reflect collaborative relationships among authors (**Figure 2B**). From **Figure 2B**, it is apparent that the most prolific authors, Auvinen A, Roobol MJ, and Tammela TLJ, published 60, 64, and 48 papers, respectively. The connections between nodes formed several tightly knit collaboration teams, with Auvinen A, Roobol MJ, and Tammela TLJ each being at the core of these teams. Auvinen A’s team was the largest. Additionally, there are several sub-networks within the network. Generally, collaboration within teams is tight, but inter-team collaboration is relatively less frequent.

The ranking of Authors’ Local Impact by H index indicates that CHINNAIYAN AM has the highest H index score, suggesting that this author’s publications have the most profound impact in the field (**Figure 2C**). The ranking of Most Relevant Authors aligns with the results of our collaboration network analysis, identifying Auvinen A, Roobol MJ, and Tammela TLJ as the top three most relevant authors in the field (**Figure 2D**). The top five authors in citation ranking are ROOBOL MJ, HUGOSSON J, LILJA H, and AUVINEN A, indicating the influential nature of their contributions (**Figure 2E**).

The burst analysis of DE KONING HJ’s authorship reveals Thompson, Ian M as the author with the highest burst strength during 2014-2016. In the last two years, emerging authors include Freedman, Matthew L, and Corey, Eva, indicating significant recent contributions in the field (**Figure 2F**).

### 3.3 Institutional collaboration network

**Figure 3:**
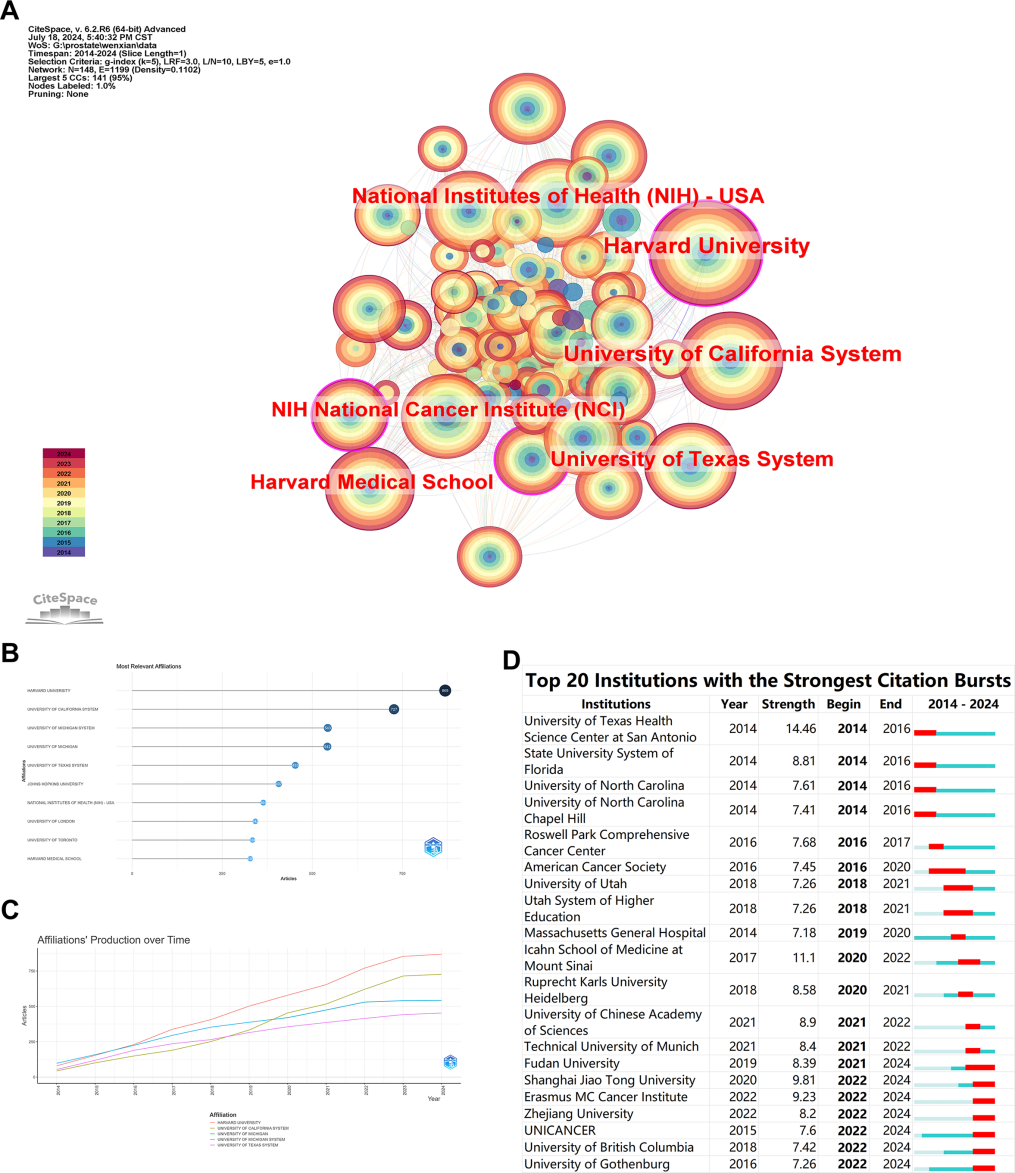
Bibliometric Visualization of Institutional Distribution in Prostate Cancer Screening. **(A)** Institutional collaboration network with the top six institutions by publication count. **(B)** Network visualization showing the most relevant institutions ranked by publication count. **(C)** Total publication output of the top five institutions over time. **(D)** Burst analysis of emergent institutions.

The institutional collaboration network reflects academic collaborations between different institutions and their research focuses. **Figure 3A** displays an institutional network comprising 148 nodes and 1199 connections. The top five institutions by publication count are HARVARD UNIVERSITY (869), UNIVERSITY OF CALIFORNIA SYSTEM (727), UNIVERSITY OF MICHIGAN SYSTEM (543), UNIVERSITY OF MICHIGAN (542), and UNIVERSITY OF TEXAS SYSTEM (453) (**Figures 3A–C**). The lines between nodes represent collaborative relationships, and the color of each node indicates the year. Among these, HARVARD UNIVERSITY shows the most frequent and closest collaborations with other institutions. The outermost purple-red nodes indicate institutions that have contributed significantly to the field in recent years, highlighting HARVARD UNIVERSITY, NATIONAL INSTITUTES OF HEALTH (NIH) - USA, and UNIVERSITY OF TEXAS SYSTEM as rapidly developing entities in this area. The burst analysis indicates that recent emergent institutions in the field include Shanghai Jiao Tong University, Erasmus MC Cancer Institute, and Zhejiang University, suggesting that domestic universities are increasingly delving into this research area (**Figure 3D**).

### 3.4 National collaboration network

**Figure 4:**
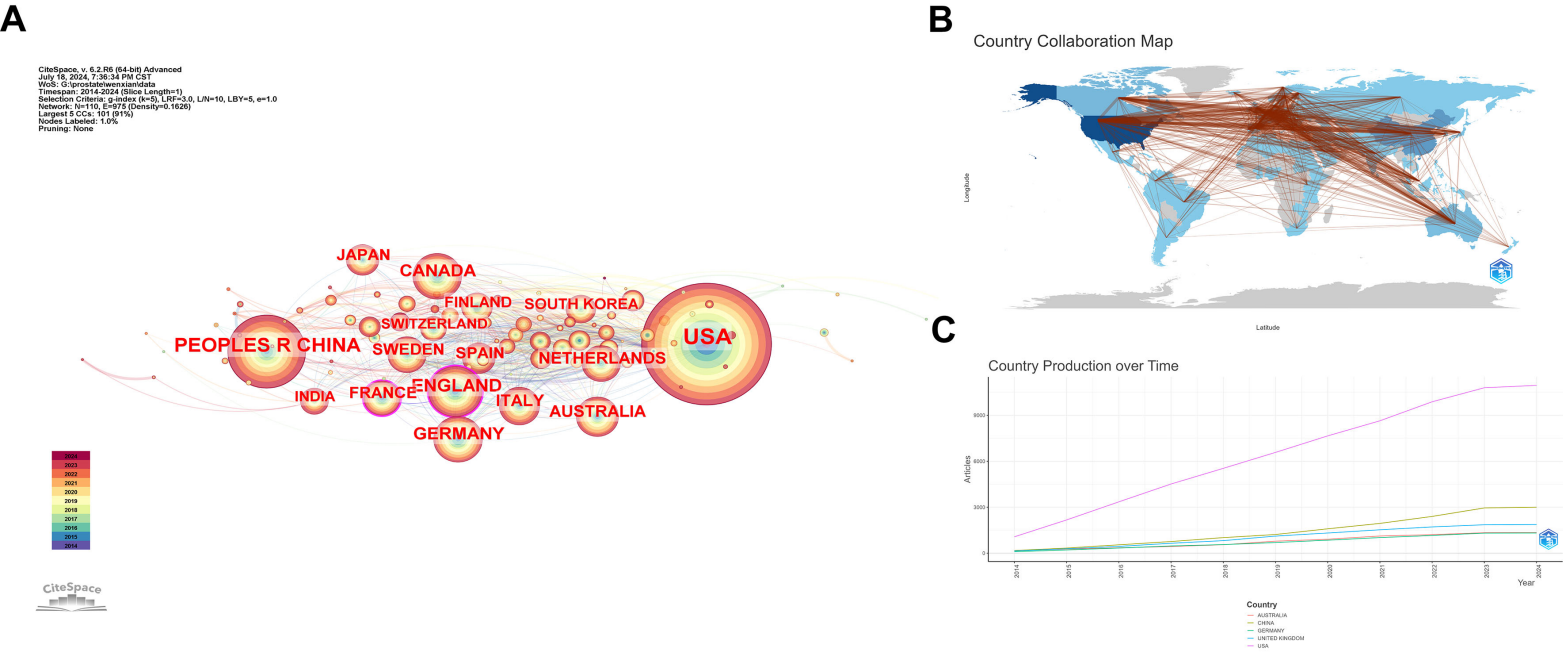
Bibliometric Visualization of Country Distribution in Prostate Cancer Screening. **(A)** National collaboration network with the top five countries by publication count. **(B)** Collaboration map with countries as nodes and collaborative relationships shown by red lines. **(C)** Total publication output of the top five countries over time.

The nodes in this network are set as countries, and **Figure 4A** reveals a network with 110 nodes and 975 connections. The top five countries by publication number are USA (2295), PEOPLES R CHINA (882), ENGLAND (425), GERMANY (394), and CANADA (367). The USA far surpasses other countries in publication numbers, indicating a deep research foundation in this field. However, as shown in **Figure 4A**, countries like France and England are experiencing rapid growth in recent research in this area. **Figure 4B**, where the color of a country node represents the number of published papers and red lines indicate collaborative relationships, shows that the USA has the strongest collaboration ties with other countries, followed by China. **Figure 4C** illustrates the publication output and speed of all collaborating authors’ countries, showing that the USA significantly leads over other countries, with China ranking second but still far behind the USA.

### 3.5 Keyword analysis

**Figure 5:**
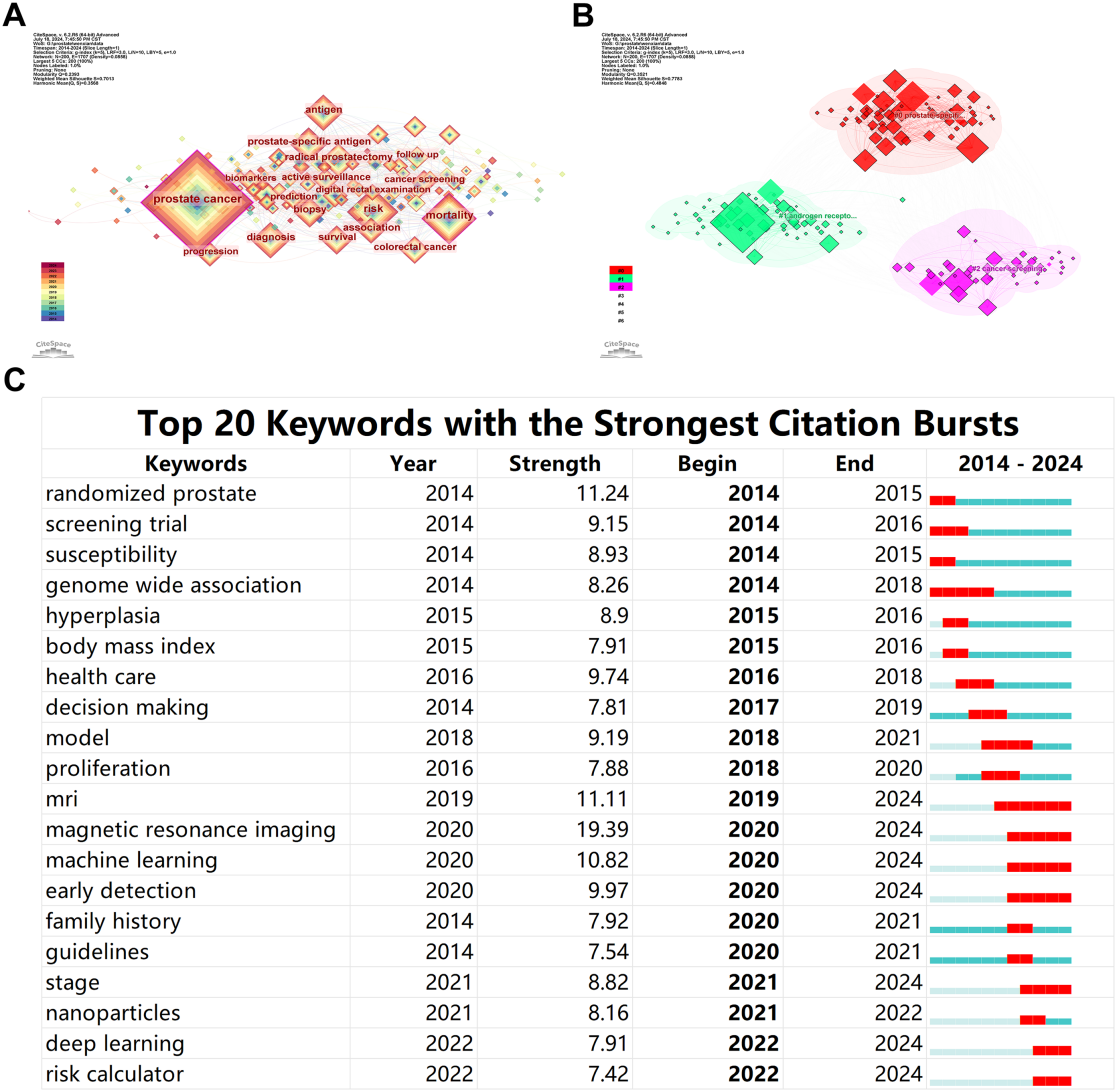
Bibliometric Visualization of Keyword Distribution in Prostate Cancer Screening. **(A)** Keyword network with the most frequent research topics. **(B)** Clustering of keywords into main research themes. **(C)** Emergent keyword analysis showing shifts in research focus.

A keyword network consisting of 200 nodes and 1707 edges was constructed, where the size of each node indicates the frequency of occurrence of each keyword in the literature. **Figure 5A** highlights that terms such as prostate cancer, mortality, prostate-specific antigen, risk, and radical prostatectomy are among the most frequently occurring keywords, representing the hot topics in this field over the past decade. Clustering these keywords revealed that they primarily fall into three clusters: #0 prostate-specific antigen, #1 androgen receptor, and #2 cancer screening (**Figure 5B**). This clustering suggests that research around these keywords is predominantly centered around these three themes.

The analysis of keyword emergence levels revealed that in the initial stages of the past decade’s research, terms like screening trial, susceptibility, and genome-wide association showed a burst of growth, indicating a focus on the genetic and hereditary factors affecting patients in prostate cancer screening. However, in recent years, the emergence of terms such as MRI (including magnetic resonance imaging), machine learning, nanoparticles, deep learning, and risk calculator indicates a shift in research focus towards the development of early non-invasive diagnostics and novel treatment methods for prostate cancer (**Figure 5C**).

### 3.6 Co-cited journals

The top 20 co-cited journals are presented in **Table 1**. The journals with the highest number of citations are European Urology (2286), The New England Journal of Medicine (2259), Journal of Urology (2104), Journal of Clinical Oncology (1812), and CA: A Cancer Journal for Clinicians (1780). CA: A Cancer Journal for Clinicians exhibits the highest centrality, at 0.11. These data indicate that these journals hold substantial academic value and influence in the field.

**Table 1:** Distribution of co-cited journals in prostate cancer research.

Journal	Frequency	Degree	Centrality
EUR UROL	2286	43	0.1
NEW ENGL J MED	2259	41	0.06
J UROLOGY	2104	40	0.06
J CLIN ONCOL	1812	40	0.08
CA-CANCER J CLIN	1780	42	0.11
JAMA-J AM MED ASSOC	1684	33	0.02
PLOS ONE	1624	46	0.1
CANCER RES	1517	72	0.15
CANCER-AM CANCER SOC	1451	37	0.02
BJU INT	1389	30	0.02
INT J CANCER	1330	34	0.05
PROSTATE	1293	35	0.04
UROLOGY	1283	25	0.02
JNCI-J NATL CANCER I	1250	31	0.04
CLIN CANCER RES	1225	56	0.07
LANCET	1220	25	0.01
BRIT J CANCER	1124	36	0.04
P NATL ACAD SCI USA	1090	60	0.06
NATURE	1083	63	0.08
CANCER EPIDEM BIOMAR	1065	34	0.08

### 3.7 Co-cited references

**Figure 6:**
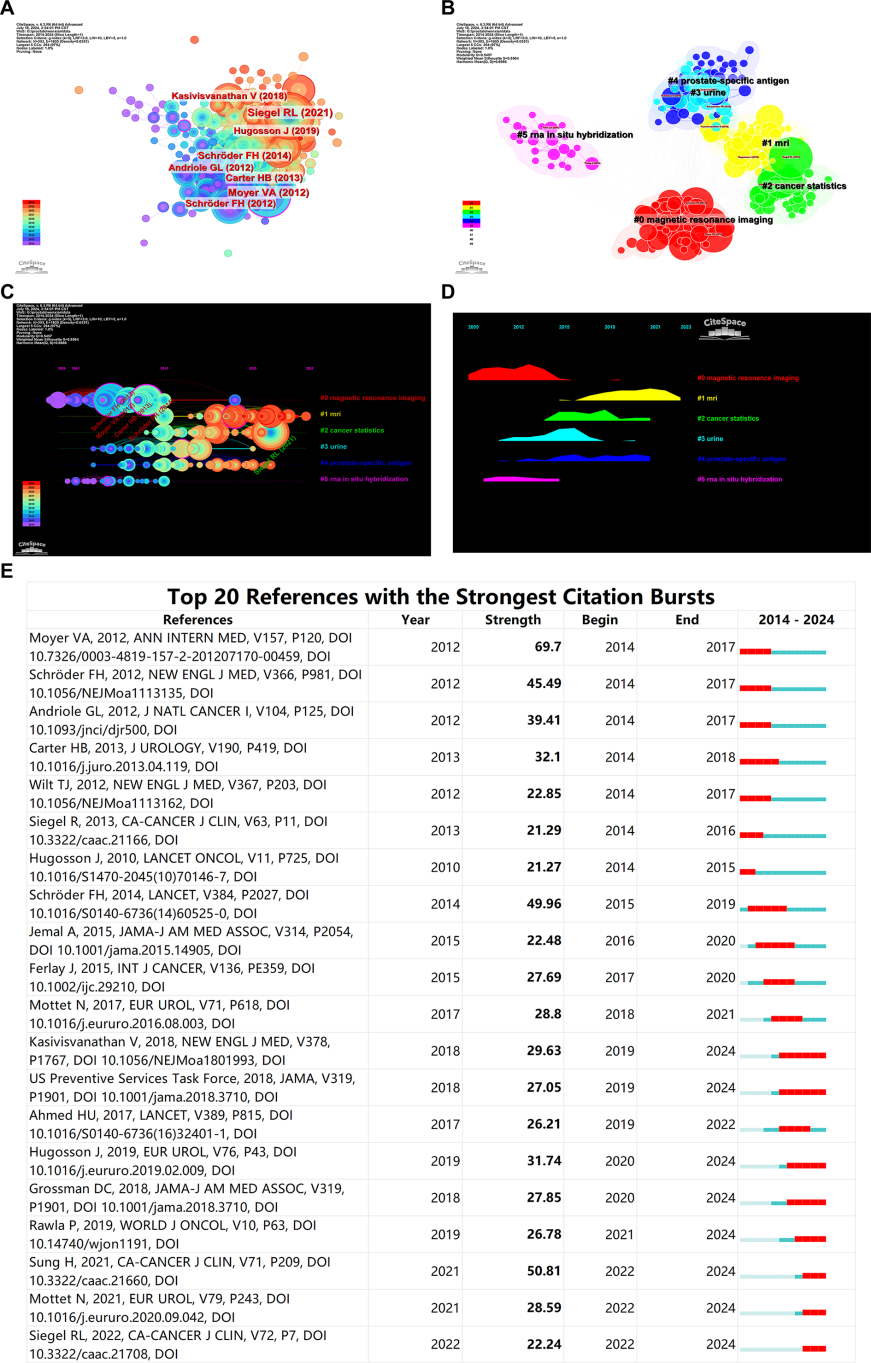
Bibliometric Visualization of Co-cited References Distribution in Prostate Cancer Screening. **(A)** Co-cited reference network showing the most co-cited references. **(B)** Keyword clusters of co-cited references. **(C)** Timeline analysis of research interest in various topics. **(D)** Popularity of research areas over time. **(E)** Burst analysis of co-cited references.

A network of co-cited references was constructed with each node representing a co-cited reference. This network comprises 303 nodes and 1605 edges, with a density of 0.0351. Based on the number of citations, the top five co-cited references are: Siegel RL (2021, 10.3322/caac.21654), Moyer VA (2012, 10.7326/0003-4819-157-2-201207170-00459), Schröder FH (2014, 10.1016/S0140-6736(14)60525-0), Carter HB (2013, 10.1016/j.juro.2013.04.119), and Schröder FH (2012, 10.1056/NEJMoa1113135), with citation frequencies of 319, 243, 200, 163, and 161 respectively (**Figure 6A**). This indicates the high authority these references hold in the field.

Key clusters identified through keyword clustering analysis of the co-cited references are distinctly categorized into five groups: #0 magnetic resonance imaging and #1 MRI (which can be combined into one category), #2 cancer statistics, #3 Urine, #4 prostate-specific antigen, #5 RNA *in situ* hybridization (**Figure 6B**). These keywords are predominantly focused on these research clusters.

Timeline analysis based on clustering reveals that research on magnetic resonance imaging (MRI) has consistently maintained high interest and remains a current hotspot. Although research on prostate-specific antigen (PSA) isn’t as heated as MRI, it has been steadily produced over the past decade. As the second most common cancer in men worldwide, cancer statistics are also a popular area of study. Urine-based prostate cancer screening research experienced a surge in popularity during 2015-2016 but has seen a gradual decline in recent years. Research including RNA *in situ* hybridization was popular from 2013 to 2015 but has faded after 2015 (**Figures 6C, D**).

**Figure 7:**
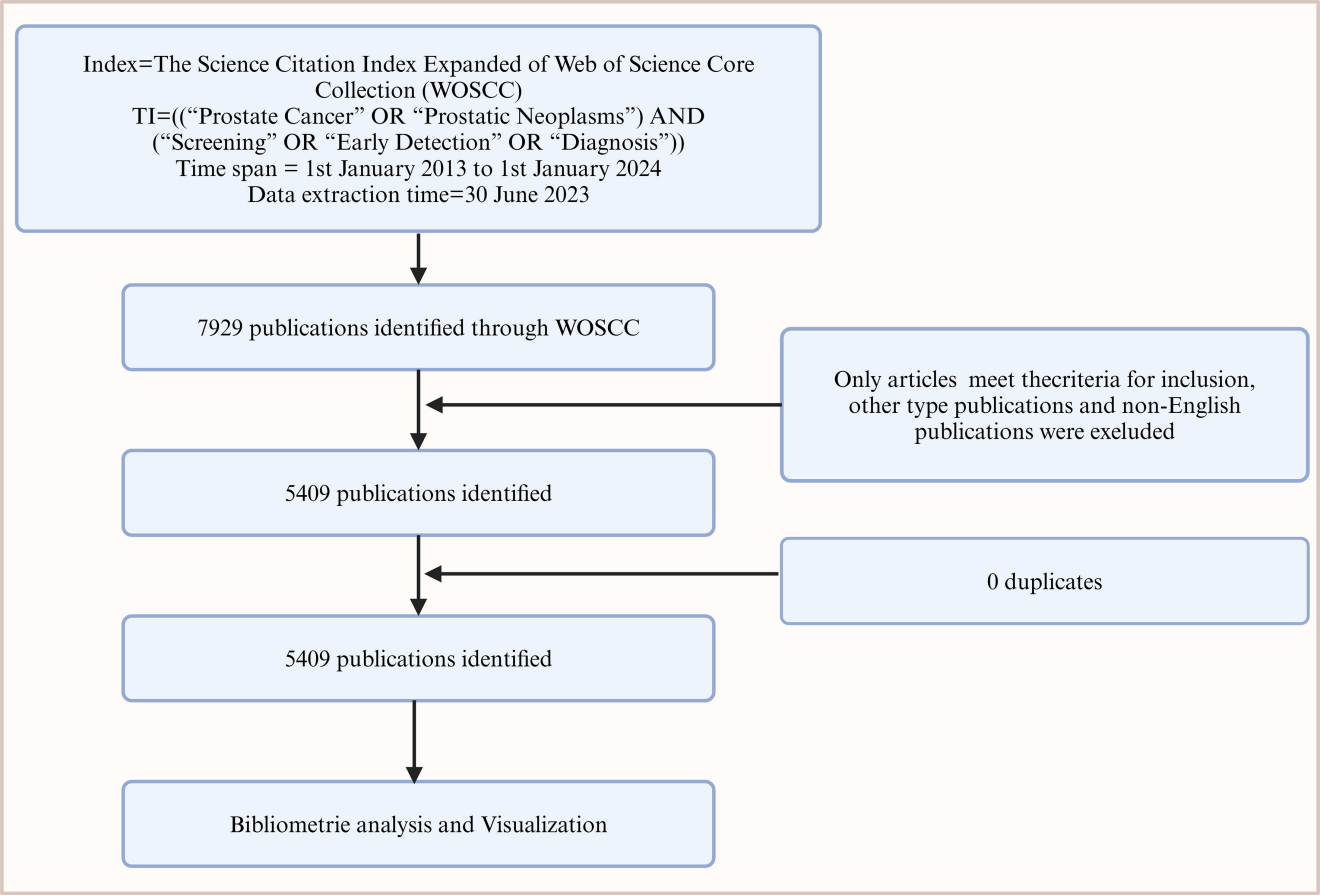
Flowchart of bibliometric analysis on prostate cancer screening literature from 2014 to 2024.

Burst analysis of co-cited references indicates that the article by Moyer VA, 2012, published in ANN INTERN MED, had the highest citation burst strength during 2012-2014, while the article by Sung H, 2021, in CA-CANCER J CLIN, has been the most frequently cited reference in the past two years (**Figure 6E**). The flowchart of this work is shown in **Figure 7**.

## 4 Discussion

This study, for the first time, systematically retrieves literature on prostate cancer screening for the period 2014-2024 using bibliometric tools and analyzes the collected 5,409 articles through bibliometric methodologies.

For over a century, the early detection and treatment of prostate cancer have been key advocacies in the medical community (12). Historically, as early as 1905, meticulous Digital Rectal Examinations (DRE) were utilized to detect the earliest signs of prostate cancer (13, 14). Over the subsequent 75 years, DRE became an essential tool for early prostate cancer screening. In the early 1980s, clinicians were initially optimistic about using the blood protein Prostate-Specific Antigen (PSA) as a screening test for the disease, driven by rising mortality rates and the poor performance of DRE (15). Although only about 25% of men with a PSA level >4.0 ng/mL were diagnosed with prostate cancer, this test still represented a significant diagnostic approach (16). Given the high prevalence of prostate cancer and its low mortality rate, serum PSA was once highly regarded globally for prostate cancer screening but later experienced a decline in popularity (17, 18).

In recent years, the advent of risk-stratified tools such as risk calculators, combined with MRI imaging analysis and deep learning applications, has introduced new methods and approaches to prostate cancer screening. These innovations have begun to transform the landscape of detection and management, offering more precise and less invasive options for identifying and treating this prevalent disease.

### 4.1 Research trends and hotspots

With the continuous development of prostate cancer screening technologies, co-word analysis and citation network analysis highlight a significant shift towards more precise and personalized screening methods. Increasing attention has been paid to genomics and molecular biomarkers, reflecting the scientific community’s efforts to identify new screening markers to reduce the risk of overdiagnosis and overtreatment.

Before the era of prostate-specific antigen (PSA) testing, many men were diagnosed with metastatic prostate cancer and succumbed to the disease; PSA testing significantly reduced prostate cancer mortality (19). However, the high rate of overdiagnosis associated with PSA-based screening led to heavy criticism and even suspension of such screenings (20). Current biomarkers, particularly PSA, are limited by their variability and the influence of non-cancerous factors such as age, prostate size, and infections, which can skew PSA levels. These limitations have significant consequences in clinical practice, where false positives can cause psychological distress and lead to unnecessary medical procedures, while false negatives might delay crucial treatment. To address these issues, future research should aim to develop more specific and sensitive biomarkers that can provide reliable information about the biological behavior of prostate tumors. This advancement could involve the exploration of molecular genetics and proteomics to identify novel biomarkers that are not only indicative of the presence of cancer but are also predictive of its aggressiveness and potential response to treatment. Additionally, the development of multi-biomarker panels and the use of non-invasive testing methods, such as liquid biopsies, could further enhance diagnostic precision and patient outcomes, minimizing the risk associated with current diagnostic limitations.

Traditionally, imaging techniques such as CT, MRI, and bone scans have been used for the diagnosis, staging, and restaging of prostate cancer. These imaging examinations, however, have many limitations. Prior to the advent of multiparametric MRI (MpMRI), clinicians faced the dilemma of “vanishing prostate cancer” – tumors undetectable by systematic biopsies but later found in prostatectomy specimens. This phenomenon, reported in up to 20% of cases, highlighted the critical limitations of traditional diagnostic pathways, particularly the high false-negative rates of conventional MRI and TRUS-guided biopsies (21). The limitations of current imaging techniques, primarily their lack of sensitivity and specificity in certain contexts, pose significant challenges in clinical settings. For instance, the high rate of false positives in prostate MRI can lead to unnecessary biopsies, which are invasive and carry potential risks such as infections and patient anxiety. Similarly, false negatives can result in missed diagnoses, allowing potentially aggressive cancers to progress undetected. The paradigm shift began with the clinical validation of MpMRI. The PROMIS trial demonstrated that MpMRI could reduce unnecessary biopsies by 27% while increasing clinically significant cancer detection by 18% compared to TRUS-guided systematic biopsies (21). Subsequent PRECISION trial data further showed that MRI-targeted biopsies alone missed 13% fewer significant cancers than standard biopsies (22). These breakthroughs addressed the diagnostic inaccuracies that previously led to overtreatment or undertreatment, each carrying its own set of clinical risks. This evidence has been codified in updated guidelines: The 2023 EAU guidelines now recommend MpMRI as a triage test before first biopsy (Grade A evidence) (23), fundamentally reshaping clinical workflows. To further enhance diagnostic precision, PSMA-targeted imaging has emerged as a synergistic partner to MpMRI. While PSMA PET excels in metastatic detection, its integration with MpMRI creates a powerful diagnostic synergy. The PRIMARY trial demonstrated that combining PSMA PET/MRI increased specificity to 94% for clinically significant cancer (vs 82% for MpMRI alone) in biopsy-naïve men (24). Molecular imaging of the prostate-specific membrane antigen (PSMA), a glycoprotein expressed 100-1000 times higher in prostate cancer cells, when combined with MpMRI, has shown remarkable accuracy in initial staging and recurrence localization. Emerging evidence supports biparametric MRI (bpMRI) as a cost-effective screening tool, particularly for high-risk populations with familial or genetic predispositions. Recent clinical trials validate the feasibility of bpMRI as a primary PSA-agnostic screening tool. In a prospective cohort of 229 biopsy-naïve men (median PSA=1.26 ng/ml), opportunistic bpMRI screening detected clinically significant PCa (csPCa) in 9.2% of participants, 38.1% of which would have been missed by PSA/DRE-based criteria (25). Critically, 54.6% of unnecessary biopsies were avoided through protocol adjustments for PI-RADS 3 lesions (6-month MRI follow-up instead of immediate biopsy). Notably, this protocol achieved clinically significant prostate cancer (csPCa) detection in 9.2% of the cohort (21/229) with a 54.6% reduction in unnecessary biopsies through deferred management of PI-RADS 3 lesions, demonstrating the potential of bpMRI as a PSA-agnostic primary screening tool (26). Photoacoustic imaging (PAI), also undergoing clinical trials, is another promising imaging technology for the future. PAI uses light excitation and ultrasound detection for high-resolution functional and molecular imaging (27). By using endogenous and exogenous contrast agents, PAI distinguishes between cancerous and benign prostate tissue with higher sensitivity and specificity than PSA testing and transrectal ultrasound (TRUS)-guided biopsies. Additionally, PAI can monitor and guide the treatment of prostate cancer. Despite these advancements, challenges persist. Standardization of PI-RADS interpretation across institutions remains inconsistent, and the technical demands of MRI-ultrasound fusion biopsies require specialized training. Future research directions should focus on integrating artificial intelligence to automate lesion classification and optimize biopsy targeting, while emerging technologies like photoacoustic imaging (PAI) – which combines light excitation and ultrasound detection – may further improve sensitivity through molecular contrast agents. These innovations collectively aim to reduce diagnostic uncertainty and usher in an era of precision oncology for prostate cancer.

The discovery of novel biomarkers for prostate cancer screening represents a pivotal advancement in the early detection and management of the disease. While Prostate-Specific Antigen (PSA) testing remains the standard method for early detection, its utility is compromised by its lack of specificity and susceptibility to influence from various non-cancerous factors such as age, acute prostatitis, ejaculation, catheterization, and certain medications. This has spurred an urgent need for more reliable non-invasive biomarkers that offer higher sensitivity and specificity. Recent research has identified several promising candidates that could revolutionize the diagnostic landscape for prostate cancer. Biomarkers such as PCA3, TMPRSS2:ERG fusion genes, and the Prostate Health Index (PHI) have emerged as significant improvements over PSA, providing more accurate distinctions between benign conditions and malignant prostate alterations (28). These advances are supplemented by innovative multi-parametric approaches that integrate genetic, epigenetic, and protein-based markers to enhance diagnostic precision. In the study by Wang R et al., potential new non-invasive biomarkers were identified by screening differentially expressed genes from the Oncomine database, including five significantly overexpressed and five significantly underexpressed genes. Further validation was conducted using qRT-PCR in prostate cancer patients and healthy donors, ultimately identifying candidate non-invasive biomarkers for diagnosing prostate cancer (29). Integrating non-invasive biomarkers and advanced bioinformatics tools into routine clinical practice faces significant challenges, including the need for upgrades in clinical pathways and laboratory capabilities, as well as the requirement for extensive clinical trials to validate their efficacy and safety. To overcome these barriers, it is crucial to upgrade medical facilities, provide comprehensive training for medical personnel, expedite regulatory approvals, and ensure the economic accessibility of new technologies while strictly adhering to data privacy laws.

The integration of nanotechnology into prostate cancer screening has significantly advanced the development of novel biomarkers, enhancing the precision of risk stratification and diagnosis. The research by Kevin M. Koo et al. demonstrated that advances in molecular subtyping and nanotechnology-based multi-omics risk stratification can refine the molecular classification of prostate cancer into clinically significant and treatable subtypes (30). Given the inter- and intra-tumor heterogeneity of prostate cancer, it is necessary to develop new nanodiagnostic techniques to rapidly, economically, and accurately identify clinically significant prostate cancer. Next-generation prostate cancer biomarkers in circulation and urine can be used for molecular subtyping of the disease, while the latest complementary nanodiagnostic platforms can enhance biomarker detection, making them promising tools for the precise management of prostate cancer (31). Specific instances of nanotechnology application in prostate cancer screening include the use of gold nanoparticle-based assays to improve the detection sensitivity of PSA levels and the development of nanoparticle-enhanced imaging techniques that provide superior specificity in identifying malignant prostate tissue (32, 33). Another promising approach involves the use of magnetic nanoparticles for the magnetic resonance imaging (MRI) of prostate cancer, offering a higher resolution and better differentiation between benign and malignant tissues (34). These innovative techniques illustrate the potential of nanotechnology to transform the traditional approaches to prostate cancer screening, enabling more accurate and earlier detection of the disease.

Consistent with our findings, keywords such as machine learning and deep learning have become increasingly important in recent years. The study by Huang Hongyuan et al. demonstrated that advancements in deep learning could further assist clinicians in accurately identifying prostate cancer cells in pathological slides, thereby achieving faster and more accurate diagnosis (35). The research by Kim Hojun et al. showed that machine learning analysis of urine multi-marker biosensors achieved over 99% accuracy in screening prostate cancer patients. The synergy between novel urinary biomarkers and machine learning analysis appears to be a highly effective collaboration. Machine learning algorithms are now widely used for risk prediction, early diagnosis, treatment selection, and prognosis monitoring of prostate cancer. For example, by analyzing massive data from multiple dimensions, including but not limited to patients’ serum PSA levels, age, race, family history, lifestyle factors (e.g., dietary habits, smoking status), medical history, and the latest medical imaging data (e.g., ultrasound, MRI scans), complex predictive models can be constructed. Through continuous learning and iterative optimization, machine learning models can evolve with new data, identifying new biomarkers and genetic variations, which could be an important component of future early warning systems for prostate cancer. This not only helps in early intervention and reduces the risk of cancer progression but also provides a scientific basis for the design of personalized treatment plans, ensuring that each patient receives the most suitable treatment strategy. The study by Bhagirath D et al. introduced a classifier developed using mechanistic learning algorithms that may non-invasively diagnose neuroendocrine differentiation induced by treatment in castration-resistant prostate cancer patients (36). This has significant implications for the clinical management of these patients. Additionally, it can predict responses to second-generation androgen receptor pathway inhibitors, which is important for the future clinical treatment of these patients.

An analysis of literature output from various countries and leading research institutions worldwide demonstrates that advanced technological infrastructure and high investment in research capabilities predominantly drive prostate cancer (PCa) screening studies in technologically advanced countries and top academic institutions. Notably, the discrepancy in research output between Western countries and Asian nations, particularly China, can be significantly attributed to the different epidemiological profiles, research funding, and strategic priorities in cancer research. Compared to Western countries, the incidence of prostate cancer is much lower in Asian countries (37). For example, in 2020, the age-standardized incidence rate (ASR) in China was 10.2 cases per 100,000 people, while in Northern European countries, the ASR was 83.4 cases per 100,000 people (38). The lower incidence reflects less perceived urgency and could lead to proportionately lower research funding and fewer initiatives targeting this disease. Globally, China has a relatively low prostate cancer mortality rate. In 2020, the age-standardized mortality rate (ASR) in China was 4.6 deaths per 100,000 people. In contrast, the Caribbean region reported the highest rate at 27.9 deaths per 100,000. This significant disparity may prompt a shift in health resource allocation towards more urgent concerns in regions with higher mortality rates (37).

Beyond epidemiological factors, systemic differences in research infrastructure such as availability of advanced diagnostic technologies, government research funding, and academic-industry collaborations significantly impact the volume and quality of research outputs. Western countries often benefit from longstanding investments in health research infrastructure, comprehensive cancer research programs, and aggressive funding strategies that attract global talent and foster innovative research. In contrast, Asian countries are rapidly developing their research infrastructures and governmental support systems to enhance their research capabilities. Despite the lower incidence of prostate cancer, China accounts for a substantial proportion of the global burden, with 8.2% of new cases and 13.6% of deaths due to its large population. This underscores the need for further attention to prostate cancer research in China. Additionally, the United States and European countries have traditionally been leaders in this field due to their substantial investments. However, the diversification of global research efforts is evident as Asian countries, notably China, make significant progress in research capabilities and outputs (39).

At the institutional level, universities such as Harvard and the University of California system in Western countries excel in prostate cancer screening research due to their robust ecosystems that include cutting-edge research facilities, strong industry links, and substantial funding. These institutions also benefit from a rich tradition of interdisciplinary research, which is crucial for innovation in medical diagnostics. Meanwhile, institutions like Shanghai Jiao Tong University and Zhejiang University in China are gaining prominence, driven by increased governmental investment in research and development, aimed at bridging the gap with Western standards and fostering innovation through enhanced international collaborations.

### 4.2 The five most frequently cited references

#### 4.2.1 Screening and prostate cancer mortality: results of the European Randomized Study of Screening for Prostate Cancer (ERSPC) at 13 years of follow-up (Fritz H Schröder, Lancet, DOI: 10.1016/S0140-6736(14)60525-0)

This study from the European Randomized Study of Screening for Prostate Cancer (ERSPC) covers a multinational long-term randomized control trial aimed at assessing the impact of PSA testing on prostate cancer mortality. Over a 13-year follow-up, PSA screening significantly reduced prostate cancer mortality by 21% compared to controls, with an adjusted reduction of 27% among those who actually participated in screening. The study confirms the effectiveness of PSA screening in reducing prostate cancer mortality but also highlights the potential risks and adverse effects, including higher incidence rates due to overdiagnosis and overtreatment (40).

#### 4.2.2 Merging new-age biomarkers and nanodiagnostics for precision prostate cancer management (Kevin M Koo, Nature Reviews Urology, DOI: 10.1016/j.eururo.2019.02.009)

This article emphasizes the importance of utilizing advanced biomarkers and nanodiagnostic technologies for precision management of prostate cancer. It explores the potential of combining next-generation prostate cancer biomarkers with nanodiagnostic platforms, heralding a new era of precise management that aims to differentiate between aggressive and non-aggressive forms of the disease and reduce unnecessary treatments. The paper also discusses the challenges and barriers to translating these technologies into clinical practice (30).

#### 4.2.3 Screening for prostate cancer: U.S. Preventive Services Task Force recommendation statement (Virginia A Moyer, Annals of Internal Medicine, DOI: 10.7326/0003-4819-157-2-201207170-00459)

This paper updates the 2008 U.S. Preventive Services Task Force (USPSTF) recommendations on prostate cancer screening, reviewing new evidence on the benefits and risks of PSA-based screening and the treatment of localized prostate cancer. Based on this evidence, the USPSTF recommends against PSA-based screening for prostate cancer in all men regardless of age (Grade D recommendation), highlighting the need for personalized decision-making in clinical practices beyond the screening itself (41).

#### 4.2.4 10-year outcomes after monitoring, surgery, or radiotherapy for localized prostate cancer (Freddie C Hamdy, The New England Journal of Medicine, DOI: 10.1056/NEJMoa1606220)

In this study, three treatment strategies for localized prostate cancer detected via PSA screening—active monitoring, radical prostatectomy, and radiotherapy—are compared. The study shows no significant difference in prostate cancer-specific mortality among the three groups after a median follow-up of 10 years, but both surgery and radiotherapy were significantly more effective at controlling disease progression and reducing the risk of metastasis compared to active monitoring (42).

#### 4.2.5 Early detection of prostate cancer: AUA Guideline (H Ballentine Carter, Journal of Urology, DOI: 10.1016/j.juro.2013.04.119)

This guideline provides a framework for urologists on the early detection of prostate cancer in men at average risk. Based on a systematic review of over 300 studies, it analyzes outcomes related to incidence/mortality rates, quality of life, diagnostic accuracy, and screening risks. Recommendations vary by age group, with a suggestion that the benefits of screening may outweigh the risks for men aged 55 to 69. The guidelines advocate for shared decision-making for men considering PSA screening in this age group (43).

Although various studies have provided different perspectives and recommendations, they consistently emphasize the necessity of considering individual variations, weighing risks against benefits, and the potential of technological innovation in implementing prostate cancer screening and treatment strategies. These discussions lay a crucial foundation for future policy formulation and clinical practice.

### 4.3 Methodological Triangulation

Our study’s strength lies in the triangulation of multiple analytical methods, each with inherent limitations. Descriptive statistical analysis provides baseline quantitative insights but may miss nuanced interactions within the research. Journal and article impact analysis, while highlighting influential studies, can bias toward older publications due to their accumulated citations and does not reflect the citation context. Trend analysis predicts future research directions but is limited by its reliance on historical data, which may not always capture fast-evolving fields accurately. By integrating these methods, our study addresses these limitations, offering a comprehensive and robust overview that enhances the validity and depth of our findings. This multi-method approach ensures a balanced exploration, effectively mapping both the current landscape and potential future developments in prostate cancer screening.

### 4.4 Limitations and future directions

This study has several limitations. First, the data were sourced exclusively from the Web of Science Core Collection, which may overlook contributions from regional or less prominent journals, potentially introducing a bias toward English-language publications and high-impact research. Second, the bibliometric analysis primarily focuses on quantitative metrics, such as citation counts and co-occurrence networks, which may not fully capture the clinical relevance or translational impact of individual studies. Notably, while the most frequent and highly cited literature often pertains to basic science, the transformative advancements in prostate cancer diagnosis, such as multiparametric magnetic resonance imaging (MpMRI), may not be proportionately reflected in citation metrics due to their more recent emergence. Future research should expand data sources to include regional databases and gray literature, ensuring a more comprehensive representation of global efforts. Additionally, integrating qualitative analyses, such as expert interviews, could provide deeper insights into the clinical application of emerging technologies like MpMRI, liquid biopsies, and artificial intelligence. Interdisciplinary collaboration will be essential to translate these advancements into practical, personalized screening strategies that minimize overdiagnosis and improve patient outcomes.

## 5 Conclusion

This study employs bibliometric tools to conduct an in-depth analysis of the advancements and trends in global prostate cancer screening research over the past decade, revealing active research hotspots and key technological developments in the field. The findings indicate a shift in research focus from traditional PSA screening to more precise and personalized methods, including advanced imaging techniques, molecular biomarkers, machine learning, and nanotechnology. The evolution of these technologies not only enhances diagnostic accuracy but also significantly reduces the risk of overdiagnosis. Future research should continue to explore and validate novel screening markers and technologies to further optimize early detection strategies for prostate cancer. Moreover, enhanced interdisciplinary collaboration will be crucial for driving further innovation and clinical application in this field. With ongoing advancements in scientific technology and deeper global collaboration, prostate cancer screening is expected to become more personalized and precise, potentially significantly improving global male health outcomes.

## Data Availability

The original contributions presented in the study are included in the article/supplementary material. Further inquiries can be directed to the corresponding author.
